# An adaptable genomic-proteomic approach to characterize cell-surface Vimentin's membrane topology and interactome

**DOI:** 10.1016/j.bbrep.2025.102242

**Published:** 2025-09-06

**Authors:** Masoud Norouzi, Christopher A. McCulloch

**Affiliations:** Faculty of Dentistry, University of Toronto, Toronto, ON, Canada

## Abstract

When found on the cell surface, Vimentin, a cytoskeletal protein, engages in multiple pathophysiological processes. Here, in human fibroblasts and endothelial cells, we used CRISPR-Cas9 to insert a hemagglutinin tag at six different loci within the *vim* gene. We then utilized tag immunodetection to define cell surface Vimentin's (csVim) membrane topology, followed by proximity biotinylation to identify its extracellular interactome; validating a subset of hits with a quantitative in vitro binding assay.

## Highlights

• Genomically inserted hemagglutinin tags shed light on cell surface Vimentin's membrane topology.
• Cell surface Vimentin's interactome is systematically characterised by proximity proteomics.
• A list of high-scoring potential cell-surface Vimentin binders and proximal proteins is provided.
• Five novel, high-affinity cell-surface Vimentin receptors/ligands are validated in vitro.

## 1 Main

Vimentin is a Type III intermediate filament protein with a well-established cytoplasmic localisation that exhibits discrete functions in cell mechanics, migration, organelle anchoring and stress-response [[1], [2], [3]]. Recently, a novel “extracellular” form of Vimentin has been described which is either localized on the surface of the plasma membrane or released into the peripheral cell environment [4]. Despite lacking a signal peptide, Vimentin can be actively secreted in response to stimuli such as cytokines and growth factors through mechanisms that aren't fully understood [5]. Golgi-mediated as well as unconventional lysosome/autophagosome-mediated secretion pathways have so far been proposed as possible routes for externalisation of Vimentin [6,7].

In numerous pathophysiological processes, cell-surface Vimentin (csVim) acts both as a (co-)receptor and as a ligand that affects angiogenesis, wound-healing, microbial infections and autoimmune diseases [8,9]. However, despite multiple, mostly coincidental discoveries of individual receptors/ligands for csVim, its membrane directionality and broader interactome are still unknown. Specifically, there is no consensus as to whether csVim is transmembrane and its orientation. Further, there are no reports so far of systematic screens for csVim's extracellular interactome despite its emerging clinical implications [[10], [11], [12], [13], [14]].

Here, we report an adaptable genomic-proteomic approach to unequivocally elucidate csVim's membrane topology and effectively identify its extracellular interactome. For this approach, and to increase confidence in the results, we conducted experiments simultaneously in two different cell lines that are known to naturally express cytoskeletal Vimentin: immortalized human gingival fibroblasts (HGF) and primary human umbilical vein endothelial cells (HUVEC).

**Fig. 1:**
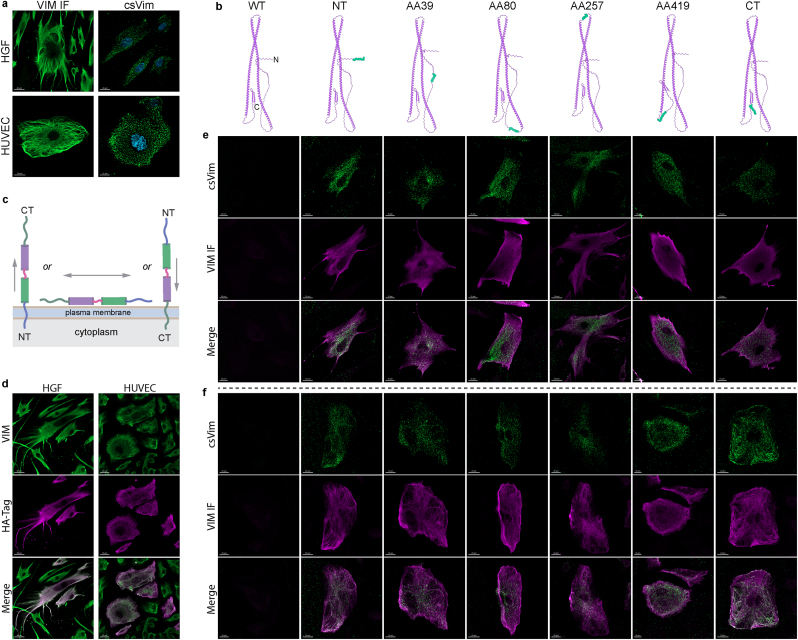
**Investigation of csVim's membrane topology. a.** Representative immunofluorescence staining of Vimentin intermediate filament (VIM IF) and csVim in wildtype (WT) HGF and HUVEC cells with an anti-VIM antibody. **b.** 3D structure representation of WT and the six HA-tag-modified versions of Vimentin, as indicated by the amino acid (AA) position of HA-tag insertion. N and C-termini are indicated for the WT, and the inserted HA-tags are marked in green. **c.** Schematic overview of the possible topologies of csVim on the cell surface. **d.** Representative VIM and HA-tag co-staining of permeabilized, non-clonally HA-tag edited, HGF and HUVEC cells with HA-tag on Vimentin's C-terminus. **e.** HA-tag-mediated immunofluorescence staining of csVim and VIM IF as indicated, in WT and HA-tag edited HGF cells presented based on the same scheme as in (**b**). **f.** Same as (**e**) but for HUVEC cells. All staining results are representative of at least 15 images. Figures in (**b**) were created in ChimeraX based on AlphaFold prediction of Vimentin's structure and the structure of HA-tag from the Protein Databank (PDB 5XCS). The scheme in (**c**) was created in Adobe Illustrator.

First, we optimised protocols and reagents to reliably detect csVim on the surface of live HGF and HUVEC cells (Fig. 1a), a uniquely challenging task due to the overwhelming labelling interference from the abundant intracellular Vimentin network that will result from unintended damage to the plasma membrane. For this, we conducted all staining steps on ice (1 °C) and prior to any fixation or permeabilization treatment.

Based on the location of unstructured regions in Vimentin's predicted three-dimensional (3D) structure from the AlphaFold protein structure database, we then designed guide RNA sequences and corresponding homology-directed repair (HDR) templates to seamlessly insert, by CRISPR-Cas9-mediated knock-in, a hemagglutinin (HA) tag at six different loci within the *vim* gene (Fig. 1b). This strategy successfully yielded six non-clonal cell lines each containing an HA-tag at indicated *vim* loci. With 10–30 % knock-in efficiency across all conditions, there were sufficient edited cell populations to conveniently conduct HA-tag immunodetection. The rationale for this approach is that if the HA-tag can be detected on the surface of non-permeabilized cells, this provides evidence that the respective domain of the Vimentin protein is localised extracellularly (Fig. 1c).

After confirming in-frame HA-tag knock-in and viable expression at both transcription and translation levels (**Extended Data** Fig. 1), we performed anti-HA-tag immunofluorescence and confocal microscopy on all edited cell lines to qualitatively determine csVim's membrane topology. It is important to note that epitope tag insertion, especially internally, might impact the biophysical properties of Vimentin. However, morphological assessment of the immunofluorescence data confirmed that all constructs maintained their functional ability to form the characteristic cytoskeletal filament network (Fig. 1d–f). Visual evaluation of the membrane topology results, provided in Fig. 1e and f, clearly indicate that csVim lacks a cytoplasmic domain and is positioned wholly on the outer surface of the cell.

Encouraged by the utility of genomically-inserted HA-tags, we then sought to leverage this exogenous epitope in a proximity biotinylation assay aimed at discovering the unknown interactome of csVim. Proximity biotinylation platforms such as BioID [15], APEX-ID [16], and antibody-guided methods [[17], [18], [19], [20]] are of considerable, recent interest because of their in situ specificity and broad coverage of potential interacting partners.

**Fig. 2:**
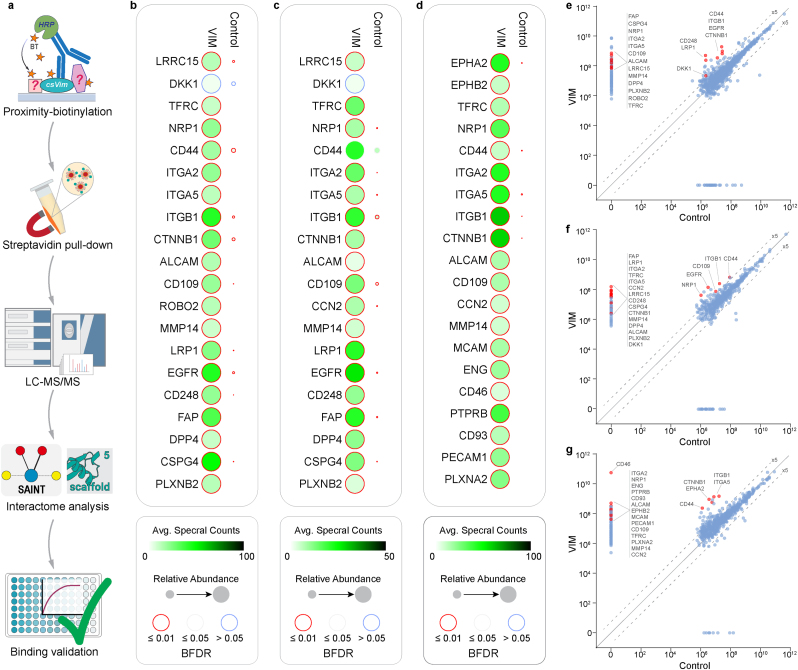
**Identification of csVim's interactome. a.** Schematic representation of the proximity biotinylation and AP-MS strategy to identify csVim's interactome. **b.** Dot-Plot representing the average (Avg.) spectral counts, the relative abundance and SAINTexpress interaction confidence scores (BFDR) for a list of 20 manually curated hits from a Vimentin-targeted AP-MS experiment in WT HGF cells (Experiment #1, see **Methods**). **c.** Same as (**b**) but HA-tag-targeted and for C-terminally HA-tag edited HGF cells; with WT cells as the Control group (Experiment #2, see **Methods**). **d.** Same as (**c**) but for HUVEC cells. **e.** Related to (**b**); scatterplot representing the relative abundance of preys in the bait (VIM) vs Control group, all respective hits in (**b**) are highlighted in red. **f.** Related to (**c**); scatterplot representing the relative abundance of preys in the bait (C-terminal HA-tagged VIM) vs Control group, all respective hits in (**c**) are highlighted in red. **g.** Related to (**d**); scatterplot representing the relative abundance of preys in the bait (C-terminal HA-tagged VIM) vs Control group, all respective hits in (**d**) are highlighted in red. Plots in (**e, f, g**) represent log10 normalised total precursor intensity values with dashed lines indicating 5x fold change. All data are representative of n = 2 independent experiments. Note that for DKK1 in (**b** and **C**), SAINTexpress did not yield a high confidence (BFDR <1 %) interaction score most likely due to its intrinsically low spectral counts [21], despite yielding a clear enrichment of 5 and 15-fold vs the Controls, respectively (see also Supplementary Tables 1 and 2). Plots in (**b**, **c**, **d**) were generated in ProHits-viz, plots in (**e**, **f**, **g**) were generated in GraphPad Prism, and the scheme in (**a**) was created in Adobe Illustrator.

Here, we used a horseradish peroxidase (HRP)-conjugated secondary antibody and a biotin-tyramide (BT) substrate in combination with primary antibodies targeting Vimentin itself or the HA-tag, followed by streptavidin affinity-purification and mass-spectrometric identification (AP-MS) of biotinylated proteins (Fig. 2a). The use of an anti-HA tag antibody here allows for a more stringent experimental set-up since the HA-tag is expressed only on a subpopulation of cells. It also enables the use of isogenic wildtype cells as the control group with identical treatment steps as the test group for AP-MS. By including orthogonal primary antibodies (anti-Vim and anti-HA-tag), we expected to increase confidence in the results and also cover potential binders that could be excluded due to epitope overlap with either antibody.

**Fig. 3:**
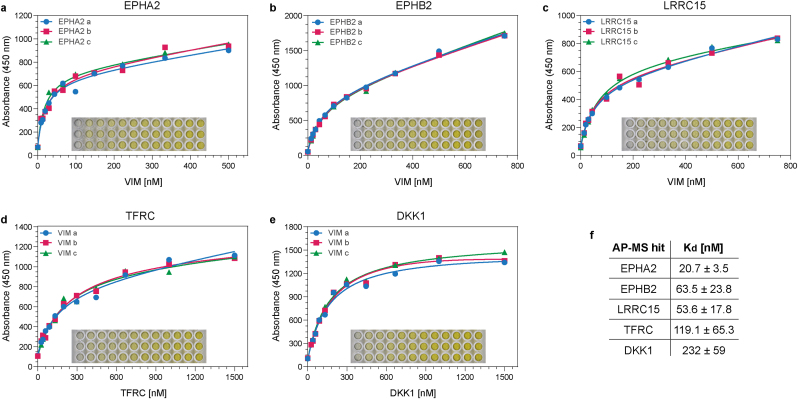
**Direct Vimentin-binding validation of selected AP-MS hits using ELISA.** Non-linear regression analysis curve fits with respective original plate images as insets for EPHA2 (**a**), EPHB2 (**b**), LRRC15 (**c**), TFRC (**d**) and DKK1 (**e**). Vimentin is used as an analyte in (**a**, **b** and **c**) and as a ligand in (**d** and **e**). **f.** Estimated equilibrium dissociation constants (*K*_d_) for the interaction of Vimentin with the hits in (**a**–**e**), as indicated. Measurements represent the mean ± standard deviation for at least 3 independent experiments (see also Supplementary Table 3).

We conducted AP-MS experiments in two configurations: one using an anti-Vim antibody on wildtype HGF cells; and one using an anti-HA-tag antibody on edited HGF and HUVEC cells (N, and C-terminal HA-tag) with respective wildtype cells as the control groups. We then examined the resulting data based on precursor (MS1) intensity enrichment and performed analysis using the Significance Analysis of INTeractome (SAINTexpress) [21] tool to identify high-confidence hits. The results are shown in Fig. 2b–g and Supplementary Table 1 and capture the high-scoring (<1 % Bayesian false discovery rate (BFDR)) and/or high-enrichment interactome of csVim on the surface of HGF and HUVEC cells, with unique and common hits between the two cell types.

Interestingly, we note that this rendition of the cell-surface proximity biotinylation method yields a remarkable AP-MS enrichment contrast between the test vs control groups—with most of the “hits” appearing as YES/NO entities i.e. non-detection in the control groups and abundant MS1 spectra in the test groups (Fig. 2b–g and Supplementary Table 2). A contributing factor for this high contrast might be the significantly smaller number of cells used in our protocol, ∼300,000 per condition compared to multiple millions in similar studies [[17], [18], [19], [20]]. Reassuringly, the great majority of enriched hits were also cell-surface or extracellular associated proteins, as confirmed by Gene Ontology term analysis (Extended Data Fig. 5**).**

In the HA-tag targeted experiment, we also noticed a marked difference between the performance of N vs C-terminally tagged groups. With only the C-terminal tag yielding high quality interactome data and the N-terminal group appearing similar to the wildtype control in HGF cells, but partially recapitulating C-terminal targeted hits at greatly reduced intensities in HUVEC cells (**Extended Data** Fig. 2). This might be due to the inadequate accessibility of csVim N-terminus in the context of proximity biotinylation and highlights the importance of bait epitope positioning in interactome analysis. Another explanation could be functional impacts from the exogenous HA-tag at the N-terminus causing subtle folding or trafficking defects that reduce its surface accessibility. Alternatively, lower N vs C-terminal knock-in efficiency can also impact the levels of HA-tag on the cell surface, and if clonally selected cell lines were used, both epitopes might yield comparable AP-MS results. Nevertheless, we proceeded with the high-quality data from the C-terminal tag experiment.

Having obtained a list of csVim-proximal proteins from AP-MS, we sought to prepare a subset of hits for in vitro binding validation assays, as proximity biotinylation broadly identifies bait-proximal proteins that may or may not be direct or high-affinity interactors [22]. We were particularly interested in identifying novel, high-affinity csVim binders as well as assessing the fidelity of our interactome identification approach. For this, we initially made a shortlist of 22 hits that met the following criteria: *i.* SAINTexpress BFDR <1 % and/or distinct enrichment in the test conditions; *ii*. established extracellular association, *iii*. potential therapeutic relevance. We then generated cDNA templates by reverse-transcription polymerase chain reaction (RT-PCR) on total RNA extracted from HGF and HUVEC cells. Based on the success of RT-PCR and pilot protein expression tests,14 final candidates were produced as recombinant proteins and purified from Expi-293T cells (Extended Data Figs. 3 and 4).

To quantitatively assess the binding of purified AP-MS hits to Vimentin and obtain approximate equilibrium dissociation constant (*K*_d_) values, we performed a reciprocal saturation enzyme-linked immunosorbent assay (ELISA) followed by non-linear regression analysis. For uniform detection and comparison of binding partners, and to avoid possible antagonist effects from an anti-Vimentin antibody, we used recombinant HA-tagged Vimentin purified from *E. coli* (**Extended Data** Fig. 4c**)**. We chose C-terminally tagged Vimentin as the interactome data were obtained using the same construct which has also been confirmed to maintain its native structural properties (Fig. 1d).

The ELISA results are summarised in Fig. 3 and Supplementary Table 3 and demonstrate that, at the indicated analyte concentration limits, three candidates, EPHA2, EPHB2 and TFRC, interact with Vimentin in both assay orientations with estimated *K*_d_ values of 20.7 nM, 65.3 nM, and 119.1 nM, respectively; and two candidates, LRRC15 and DKK1, interact with Vimentin in at least one assay orientation with estimated *K*_d_ values of 53.6 nM and 232 nM, respectively. Overall, validating 5 out of the14 tested AP-MS hits as previously unknown, direct csVim binders with nanomolar affinity.

The high-scoring csVim binding candidates (Fig. 2, Supplementary Table 1), as well as the validated subset presented above, represent a valuable resource for follow-up studies on the pathophysiological functions of extracellular Vimentin. For example, EPHA2 and EPHB2 are both receptor tyrosine kinases, and csVim was recently shown to mimic angiogenic ligand functions for another receptor tyrosine kinase the vascular endothelial growth factor receptor (VEGFR) [7]. In another study, cytoskeleton-associated protein 4 (CKAP4), a known receptor for DKK1 [23], was shown to specifically co-purify with Vimentin [24]. TFRC and LRRC15 have both been implicated as receptors for the SARS-CoV-2 Spike protein [25,26], a role that was also discovered for csVim [27]. Such background information can guide the design of follow-up studies aiming to explore the functional significance of these novel csVim interactions.

In conclusion, we have developed and validated an adaptable genomic-proteomic approach for elucidating the membrane topology and identifying the extracellular interactome of cell-surface Vimentin. Our study does have limitations which should be taken into account when interpreting its findings. For example, the membrane topology data were obtained from Vimentin variants with an exogenous HA-tag that might impact its structural integrity and physiological properties. Notably, morphological observations indicated that all tagged variants were capable of functional filament formation. The binding affinity data were also obtained using recombinant Vimentin-HA that might not fully replicate affinity profiles from wildtype Vimentin on the cell surface. Finally, the csVim interactome identified here might be influenced by the cell types and culture conditions used [28]; however, the methodology is adaptable and can be employed to study csVim in other contexts.

We anticipate that this approach would find utility in characterising the structure and function of orphan receptors and any cytoplasmic proteins that have moonlighting presence on the cell-surface. We also anticipate that the repertoire of potential and validated csVim-binding proteins presented in this work would facilitate further investigation and offer new opportunities for the development of biologic interventions for csVim-associated health conditions ranging from cancer [10] to viral infections [12] to fibrosis [14].

## 2 Methods

### 2.1 Mammalian cell lines and growth conditions

hTERT-immortalized Human Gingival Fibroblast (HGF) cells were obtained from Applied Biological Materials (#T0026) and grown in Dulbecco's Modified Eagle Medium (DMEM) containing 10 % fetal bovine serum (Wisent). Primary Human Umbilical Vein Endothelial Cells (HUVEC) from pooled donors (#C-12203) were obtained and grown in Endothelial Cell Growth Medium (#C-22010) from PromoCell. Both cell lines had been confirmed by suppliers to be free of mycoplasma contamination. Cells were always grown in a tissue culture incubator set to 37 °C and 5 % CO_2_.

### 2.2 CRISPR-Cas9 knock-in of HA-tag at *vim* loci

The cDNA sequence of Vimentin was obtained from the Ensemble database (ENST00000544301.7). Vimentin's predicted 3D structure was obtained from the AlphaFold protein structure database (AF-P08670-F1-v4), visualised in ChimeraX [29] (v1.9), and 6 unstructured regions across the protein were selected for HA-tag knock-in (Fig. 1b). Guide RNA (gRNA) and corresponding HA-tag HDR donor oligonucleotide sequences were designed using the TrueDesign Genome Editor (Invitrogen) and ordered for synthesis from Integrated DNA Technologies (IDT, Alt-R CRISPR-Cas9 crRNA and tracrRNA, and Alt-R HDR Donor Oligo). All gRNA and HDR oligo sequences are provided in Supplementary Table 4.

The Cas9 enzyme was purified in-house after expression using the pET15 SP-Cas9 plasmid (Addgene, #62731) in *E.coli* BL21(DE3) cells (NEB), and stored in a solution containing 10 mM tris (pH 7.8), 250 mM NaCl, 0.5 mM Dithiothreitol (DTT), and 50 % glycerol. gRNA complexes were assembled as follows: 2 μl of tracrRNA and 2 μl of respective crRNA stocks, each resuspended to 200 μM in Duplex Buffer (IDT #11010301), were mixed and hybridised by heating to 95 °C for 5 min and cooling to room temperature (RT) for 10 min. Ribonucleoprotein (RNP) complexes were assembled by mixing 4 μl of the gRNAs with 5 μl of the Cas9 protein (68 μM stock) and incubating at RT for 15 min.

RNP delivery was performed using the Cell Line Nucleofector Kit V on an Amaxa Nucleofector II instrument (Lonza). For each transfection reaction, early passage (P4) HUVEC (1.2 × 10^6^ cells) or HGF (6 × 10^5^ cells) cells were pelleted, washed once in 1x phosphate buffered saline (PBS), and resuspended in 102 μl of electroporation mixture containing: 91 μl of Nucleofector Solution V, 2 μl of HDR donor oligo (200 μM stock in IDT Duplex Buffer), and 9 μl of the RNP complex. Electroporation was conducted using the programs A034 for HUVEC and A024 for HGF cells. The cells were then recovered in a final volume of 8.5 ml growth media containing 270 nM of IDT's Alt-R HDR Enhancer V2 and allowed to grow overnight in a T75 flask (Corning). 18 h after transfection, growth media was aspirated, a PBS wash was performed, and fresh media was added to the cells. The edited cells were grown for 5–7 days to confluence and expanded for downstream analysis, saving frozen stocks at the first passage post-transfection. All analysis was performed before the edited cells reached passage 6 post HA-tag knock-in.

### 2.3 RT-PCR

Approximately 1.5 × 10^6^ cells were subjected to total RNA extraction with the RNeasy mini kit (Qiagen) and the resulting eluates were used as templates for cDNA synthesis with the iScript cDNA synthesis kit (Bio-Rad) or the ProtoScript II Reverse Transcriptase kit (NEB). Gene-specific forward and reverse primers (targeting only the ectodomain for receptors) were designed based on the CCDS database (https://www.ncbi.nlm.nih.gov/projects/CCDS/CcdsBrowse.cgi) and used with the Q5 High-Fidelity 2X Master Mix (NEB) to PCR-amplify cDNA sequences. All cDNA CCDS links and primer sequences are provided in Supplementary Table 5. Primer sequences for HA-tag knock-in verification are provided in Supplementary Table 4.

### 2.4 Western blot

For western blotting (WB) of whole cell lysates, approximately 2 × 10^6^ cells were lysed in 350 μl of lysis buffer (50 mM tris (pH7.8), 300 mM NaCl, 1 % NP40, 0.5 % Triton-X100, 1 mM DTT, 0.1 % sodium dodecyl sulfate (SDS), 1 mM Ethylenediaminetetraacetic acid (EDTA), and protease inhibitors (Roche #11836170001)). After centrifugation at 5000×*g* for 5 min, 15 μl of the cleared lysate was subjected to SDS-polyacrylamide gel electrophoresis (SDS-PAGE) and WB. The blots were probed with antibodies against Vimentin (Proteintech, #10366-1), HA-tag (CST, #C29F4), and GAPDH (Proteintech, #60004).

### 2.5 Immunofluorescence and confocal microscopy

Vimentin rabbit polyclonal antibody (pAb) was from Proteintech (#10366-1) and used at 1 μg/ml. HA-tag rabbit monoclonal antibody (mAb) was from Cell Signalling Technology (CST, #C29F4) and used at 132 ng/ml. HA-tag mouse mAb was from Proteintech (#66006-2) and used at 1 μg/ml. Alexa Fluor-conjugated 2° antibodies were from Invitrogen (AF-488: #A11034; AF-647: #A21235) and used at 4 μg/ml. Antibodies were always diluted in 1 % bovine serum albumin (BSA) in PBS, and 300 μl volumes were added per sample.

For all immunofluorescence experiments, approximately 30,000 HGF/HUVEC cells were grown overnight in each well of a 4-well chambered cover glass (Cellvis, #C4-1.5H–N) precoated with Fibronectin. On the next day at around 70 % confluence, the dishes were placed on ice, growth medium was aspirated, and the cells were washed once with ice-cold PBS. For the detection of cell-surface Vimentin or HA-tag, the cells (non-fixed/permeabilized) were incubated with Vimentin rabbit pAb or HA-tag rabbit mAb, respectively, for 1 h on ice. The antibody mixture was removed, and the wells were washed once with ice-cold PBS. The cells were then incubated with the AF-488-conjugated 2° Ab (goat anti-rabbit) for 30 min on ice, followed by x3 washes with ice-cold PBS. The live-stained cells were fixed with 4 % Formaldehyde for 15 min at RT and the fixation solution was aspirated. The fixed cells were permeabilized with 0.2 % Triton X-100 for 5 min at RT followed by one final wash with PBS.

For the detection of cytoskeletal/intracellular HA-tagged Vimentin, the fixed and permeabilized cells were incubated with the HA-tag mouse mAb for 30 min at RT followed by one PBS wash. The cells were then incubated with the AF-647-conjugated 2° Ab (goat anti-mouse) for 30 min at RT and washed three times with PBS. The cells were fixed again for 5-min at RT and washed with PBS for one final time. 1 ml of PBS was added to each well and the slides were sealed with parafilm and stored at 4 °C until imaging. The same procedure was followed for the staining of wildtype cytoskeletal Vimentin, except the Vimentin rabbit pAb and AF-488-conjugated 2° Ab (goat anti-rabbit) were used.

Imaging and analysis were performed in The Collaborative Advanced Microscopy Laboratories of Dentistry (CAMiLoD). Confocal microscopy was performed on an LSM800 Zeiss confocal laser scanning microscope using a 40x objective lens. Confocal images were visualised and assessed on the Imaris image analysis software (V. 10.1).

### 2.6 Proximity biotinylation

Two configurations of proximity biotinylation were conducted on different dates (one year apart): Experiment #1: performed on wildtype HGF cells using the Vimentin 1° Ab and an HRP-conjugated 2° Ab in the test condition, and only the 2° Ab in the control condition; Experiment #2: performed on Vimentin N-and-C-terminally HA-tag edited HGF and HUVEC cells as the test condition, and respective wildtype cells as the control condition, using the HA-tag 1° Ab and an HRP-conjugated 2° Ab across all conditions.

For proximity biotinylation, 3 × 10^5^ HGF/HUVEC cells were seeded in biologically independent duplicates per condition overnight in each well of a 6-well tissue culture plate (Corning, # 353046). On the next day at around 90 % confluence, the plates were placed on ice and media was aspirated followed by a 2 ml ice-cold PBS wash. Primary antibodies were diluted in 1 % BSA and added in 1 ml volumes/well as follows:

Experiments #1, Vimentin rabbit pAb (Proteintech #10366-1, raised against full-length protein with unknown epitope(s)) at 1 μg/ml for the test condition, or only 1 % BSA for the control condition. Incubated on ice for 1 h, followed by four washes each with 2 ml ice-cold PBS.

Experiments #2, HA-tag rabbit mAb (CST, #C29F4) at 132 ng/ml. Incubated on ice for 1 h, followed by one wash with 3 ml ice-cold PBS.

HRP-conjugated secondary antibody (Abcam, #ab205718) was diluted to 2 μg/ml in 1 % BSA and 1 ml was added per well in all conditions. The plates were incubated for 30 min on ice, followed by three washes each with 3 ml ice-cold PBS. Then, 1 ml/well of a biotinylation solution containing 500 μM Biotinyl tyramide (Sigma Aldrich, #SML2135) and 980 μM H_2_O_2_ in PBS was added and incubated at RT for exactly 3 min, followed by four washes each with 2 ml PBS. Finally, the cells were lysed with 1 ml/well of lysis buffer (50 mM tris (pH7.8), 300 mM NaCl, 1 % NP40, 0.5 % Triton-X100, 1 mM DTT, 5 % glycerol, and protease inhibitors (Roche #11836170001)), and total lysates were flash-frozen and stored at −80 °C until use.

### 2.7 Streptavidin pull-down

For purification of biotinylated proteins, 400 μg of Streptavidin magnetic beads (NEB, #S1420S) was added to each 1 ml lysate from proximity biotinylation and incubated on a shaker at 4 °C for 2 h. The flowthrough was aspirated, and the beads were washed twice each with 1 ml of PBS supplemented with 0.5 M NaCl, 0.05 % Tween-20, and 0.32 M urea. The beads were subjected to another three washes each of 1 ml PBS and stored dry at −80 °C until use.

### 2.8 Liquid chromatography tandem mass spectrometry (LC-MS/MS)

All sample preparation, LC-MS/MS data collection, and MS data searches were carried out at the SPARC BioCentre at The Hospital for Sick Children (Toronto, ON, Canada). Samples were reduced with 10 mM DTT at 60 °C for 1 h, alkylated with 20 mM iodoacetamide at RT for 45 min in the dark, and digested with Trypsin (2 μg, Pierce) at 37 °C overnight. Peptides were lyophilized on a SpeedVac instrument (Thermo Scientific), desalted using a C18 ZipTip (Millipore), and lyophilized again before resuspension in Buffer A (0.1 % formic acid, 2 % acetonitrile). Samples were analysed by LC-MS/MS on an EASY-nLC 1200 liquid chromatography system with a 1-h analysis duration, and an Orbitrap Exploris 480 mass spectrometer (Thermo Fisher Scientific). For the LC portion of the analysis, an 18-min linear gradient at 3–20 % was run from Buffer A to Buffer B (0.1 % formic acid, 80 % acetonitrile), followed by a 31-min linear gradient at 20–35 % of Buffer A to Buffer B, a 2-min ramp to 100 % Buffer B and 9 min hold at 100 % Buffer B, all at a flow rate of 250 nl/min. Samples were loaded into a 75 μm × 2 cm Acclaim PepMap 100 trap column followed by a 75 μm × 50 cm PepMap RSLC EASY-Spray analytical column filled with 2 μM C18 beads (Thermo Fisher Scientific). MS1 Orbitrap resolution was set to 60,000 for a scan range of *m*/*z* 400–1600 with a maximum ion injection time (IT) of 150 ms, a radio frequency (RF) lens of 30 %, a normalised automatic gain control (AGC) target of 300 %, and dynamic exclusion set to 8 s. Isolation for MS2 scans was performed in the quadrupole mass filter with an isolation window of *m*/*z* 2.5. MS2 scans were performed in the Orbitrap at a resolution of 15000, a maximum IT of 50 ms, and higher-energy collisional dissociation (HCD) with a normalised collision energy of 30 %.

Tandem mass spectra were analysed using the Proteome Discoverer software (v2.5.0.400) and fragment lists were searched against the human proteome on the UniProt database (Uniprot_UP000005640, downloaded Sep 15, 2020; 74854 entries) using the Sequest search engine (Thermo Fisher Scientific, version IseNode in Proteome Discoverer) with the following parameters: parent mass tolerance: 50 ppm; fragment mass tolerance: 0.020 Da; digestion enzyme: Trypsin; maximum missed cleavages: 3; fixed modifications: carbamidomethylation of cysteine; variable modifications: oxidation of methionine, deamidation of asparagine and glutamine, and acetylation of the protein N-terminus.

Scaffold (v5.3.3, Proteome Software) was used to validate MS/MS based peptide and protein identifications. Peptide identifications were accepted if they could be established at greater than 95.0 % probability by the Percolator [30] posterior error probability calculation. Protein identifications were accepted if they could be established at greater than 99.0 % probability by the ProteinProphet [31] algorithm and contained at least 2 identified peptides.

### 2.9 AP-MS data analysis and confidence scoring

To assess relative prey abundance enrichment in AP-MS Test vs Control groups, Normalised Total Precursor Intensity (NTPI) values for all detected proteins in each experiment were obtained from Scaffold (v5.3.3) (Supplementary Table 2) and visualised on a Condition-Condition scatter plot using the ProHits-viz analysis and visualisation webtool (https://prohits-viz.org/) (Fig. 2e–g, and **Extended Data** Fig. 2).

SAINTexpress [21] interaction confidence scoring was performed on the FragPipe proteomics data analysis platform (v22.0; Nesvizhskii lab, Ann Arbor, Michigan) connected to the MSFragger [32] (v4.1) search engine and the IonQuant [33] (v1.10.27) label-free quantification (LFQ) program. For this, Thermo.raw data files were used as input and processed using the “Default” workflow on FragPipe. The human reference proteome FASTA database (Uniprot_UP000005640, downloaded Apr 03, 2025) was set as the sequence database (166866 entries, 83433 (50 %) decoys). MSFragger was run selecting the “closed search default configuration” with the following search parameters: precursor mass tolerance: 20-20 ppm; fragment mass tolerance: 20 ppm; calibration and optimisation: mass calibration, parameter optimisation; digestion enzyme: Trypsin; maximum missed cleavages: 2; variable modifications: oxidation of methionine and acetylation of the protein N-terminus; fixed modifications: carbamidomethylation of cysteine. MSFragger search results were further filtered by Percolator [30] and ProteinProphet [31] using the Validation tool, followed by MS1 LFQ using the IonQuant tool. Finally, SAINTexpress analysis was performed on the “Downstream” tab with the following settings: max number of replicates:10; number of virtual controls: 100.

Full SAINTexpress spectral count and MS1 intensity-based analysis reports for all three AP-MS experiments are provided in Supplementary Table 1. For generation of Dot-Plots (Fig. 2b–d), a manually curated list of 20 high-scoring (BFDR <1 %) and/or highly enriched hits per experiment (incorporating all 22 initially shortlisted candidates, where present) was analysed and plotted on the ProHits-viz webtool (https://prohits-viz.org/). ShinyGO gene-set enrichment tool [34] was used for Cellular Component GO term analysis of top-scoring AP-MS hits (**Extended Data** Fig. 5).

### 2.10 Cloning of Vimentin and AP-MS candidates

For all cloning experiments, the NEBuilder HiFi DNA Assembly Master Mix (NEB) was used following manufacturer's instructions. For Vimentin-HA, the coding sequence for human Vimentin (UniProt P08670) with a C-terminal HA-tag was codon-optimised for *E. coli* expression, synthesised as a gene block (Twist Bioscience), and cloned into a pET24b plasmid backbone (Novagen, originally Addgene #111702). The SpyCatcher-Apex chimera was prepared essentially as described in Norouzi et al. [35], except the SpyCatcher03 [36] was used here.

All cDNAs for AP-MS candidates (except for CSPG4, which did not amplify successfully) were obtained using RT-PCR as described above. The cDNAs were first cloned into the pT7CFE1 expression vector with an N/C-terminal SpyTag003 [36] (depending on the ectodomain position) for expression verification using the 1-Step Human Coupled IVT Kit (Thermo Scientific #88882) followed by WB detection using SpyCatcher-Apex (**Extended Data** Fig. 4a). Following this initial test, all cDNAs were then re-cloned into a Clonetech N1 vector backbone (Addgene #58029) with a human Trypsinogen-2 secretion signal [37] and an N-terminal x6 His-tag, whilst keeping the N/C-terminal SpyTag003 [36]. Cloned sequences were always confirmed by Sanger sequencing. All final coding frame amino acid sequences are provided in Supplementary Table 6, note that based on the UniProt database some cDNAs were known isoforms or contained natural variants, as indicated in Supplementary Table 5.

### 2.11 Recombinant protein expression and purification

For Vimentin-HA, an overnight BL21 (DE3) *E. coli* (NEB) culture was diluted 1:100 in 1 L of Luria-Bertani broth and allowed to grow at 37 °C with shaking at 220 RPM until mid-exponential phase (OD600 of 0.7). Protein expression was induced by the addition of 800 μM Isopropyl β-d-1-thiogalactopyranoside (Sigma-Aldrich), and the culture was grown for 5 h at 35 °C before harvesting the cells. The cell pellet was first lysed with sonication in 30 ml of lysis buffer (20 mM sodium phosphate pH 7.4, 0.5 M NaCl, 1 mM DTT, 100 μg/ml each of lysozyme and DNase I (Sigma-Aldrich), and protease inhibitors (Roche)) and spun at 20,000×*g* for 45 min, discarding the supernatant. The resulting pellet was washed twice each with 30 ml of wash buffer (50 mM tris pH 7.8, 0.5 M NaCl, 1 % Triton X-100, 1 mM DTT), and one final time with 30 ml of 20 mM tris pH 7.8, spinning at 20,000×*g* for 45 min after each wash. The final pellet was resuspended in binding buffer (50 mM tris pH 7.8, 8 M urea, 1 mM DTT) and purified with a HiTrap Q XL anion exchange column (Cytiva) on an NGC Quest 10 Plus System (Bio-Rad); eluting the pure protein in binding buffer containing 1 M NaCl. The eluate was buffer exchanged on a PD10 desalting column (Cytiva) into storage buffer (5 mM sodium phosphate pH 7.8, 1 mM DTT) and aliquots were flash frozen and stored at −80 °C.

For recombinant expression of AP-MS candidates, Expi293F cells (Gibco) were grown in Expi293 Medium (Gibco) according to manufacture's instructions. After an initial expression test (**Extended Data** Fig. 4b), selected Plasmids were transfected into 25 ml culture volumes using the ExpiFectamine 293 Transfection Kit (Gibco) per manufacturer's protocol. Conditioned media containing secreted recombinant proteins were harvested 5 days post-transfection, spun at 4000×*g* for 30 min, and filtered through a 0.2 μm syringe filter (Millipore). The resulting filtrates were supplemented with 2 ml of 2 M NaCl and 800 μl of 1 M sodium phosphate pH 7.8, bringing the final volume to ∼32 ml. For protein purification, 100 μl of His60 Ni Superflow Resin (Takara) was added to each sample, and incubated overnight on a shaker at 4 °C. On the next day, the resin was captured on a custom-made mini column attached to a vacuum pump. The resin was then washed with 4 ml of PBS, followed by 4 ml of 5 mM imidazole in PBS. Proteins were eluted by brief centrifugation in 3 × 250 μl volumes of elution buffer (PBS containing 250 mM imidazole). Final products were buffer-exchanged into PBS using centrifugal concentrators (Millipore), and aliquots were flash-frozen and stored at −80 °C.

Protein concentrations were measured on a NanoDrop-1000 spectrophotometer (Thermo Scientific) using absorbance at 280 nm and theoretical extinction coefficients. Protein quality was always verified by SDS-PAGE and Coomassie staining (**Extended Data** Fig. 4c).

### 2.12 ELISA binding validation and data analysis

For all ELISA assays, 96-well polystyrene microplates (Corning #9017) and the 1-Step Ultra TMB ELISA substrate solution (Thermo Fisher) were used. All wash steps were conducted on a BioTek ELx50 automatic microplate washer set to 5 cycles of 300 μl washes per well. The wash buffer was PBS containing 0.05 % Tween-20, except for the final wash which was with PBS only. Unless indicated otherwise, reagents were always dispensed on ice using an 8-channel pipette to minimise drying of the wells. All incubations were performed at RT with gentle shaking.

For ligand immobilization, wells were coated with 38 μl/well of indicated ligands at 10 μg/ml in PBS for 1 h. Three extra wells per ligand were also coated with PBS only as the negative control. To block the uncoated surfaces, 280 μl/well of blocking buffer (PBS containing 5 % milk powder and 0.02 % Tween-20) was added directly to each well and incubated for 1 h. A wash step was performed, and 35 μl/well of analytes at indicated concentrations in binding buffer (PBS containing 0.1 % BSA and 0.02 % Tween-20) were added. Analyte incubations were performed for 30 min if Vimentin-HA was the analyte, or for 1 h if AP-MS candidates were the analytes. A wash step was performed, and 35 μl/well of primary antibody in binding buffer was added as follows: HA-tag (CST, #C29F4) at 132 ng/ml if Vimentin-HA was the analyte; or SpyTag (Bio-Rad #HCA406) at 666 ng/ml if AP-MS candidates were the analytes. A wash step was performed, and 35 μl/well of HRP-conjugated 2° Ab (Abcam, #ab205718) at 2 μg/ml in binding buffer was added, incubating for 30 min. A wash step was performed, followed by a final wash step with PBS only.

For signal development, 40 μl/well of the TMB substrate was added and incubated for 3 min, followed by 40 μl/well of 2 M sulfuric acid to quench the reaction. Absorbance measurements were immediately taken at 450 nm on a Multiscan FC microplate photometer (Thermo Scientific).

To obtain approximate equilibrium dissociation constant (*K*_d_) values, non-linear regression (curve fit) analysis with the inbuilt Saturation Binding equation (total binding, one-site) in GraphPad Prism (v10.4.2) was used. All ELISA assays with a positive binding result were conducted in technical triplicates and independently repeated a minimum of three times for at least one assay orientation. Results for all ELISA experiments with accompanying curve fit analysis reports, where applicable, are provided in Supplementary Table 3. It should be noted that for EPHA2, EPHB2 and TFRC, where clear binding was observed in both assay orientations, the reported *K*_d_ reflects affinity in one orientation (EPHA2/B2 as ligand, and TFRC as analyte). As under the described experimental conditions, the obtained binding curves in the opposite assay orientation did not fit a simple one-site binding model.

## Author contributions

CM and MN conceptualised the study; MN developed all methodology, conducted all experiments, analysed the data and wrote the manuscript with help and supervision from CM; CM was responsible for funding acquisition.

## Declaration of competing interest

The authors declare the following financial interests/personal relationships which may be considered as potential competing interests: Christopher McCulloch reports financial support was provided by 10.13039/501100000024Canadian Institutes of Health Research. If there are other authors, they declare that they have no known competing financial interests or personal relationships that could have appeared to influence the work reported in this paper.

## Data Availability

All data is already presented within the article and supporting information files.
