# Supplementary figures and images for: An adaptable genomic-proteomic approach to characterize cell-surface Vimentin's membrane topology and interactome
Source: Biochem Biophys Rep. 2025 Sep 6;44:102242. doi: 10.1016/j.bbrep.2025.102242 (PMC12447900; doi:10.1016/j.bbrep.2025.102242)

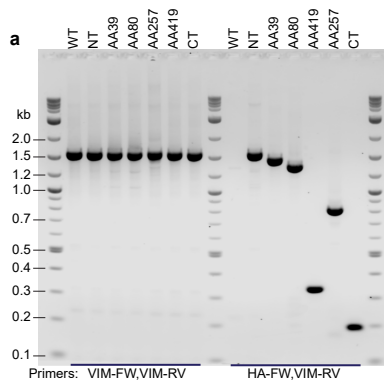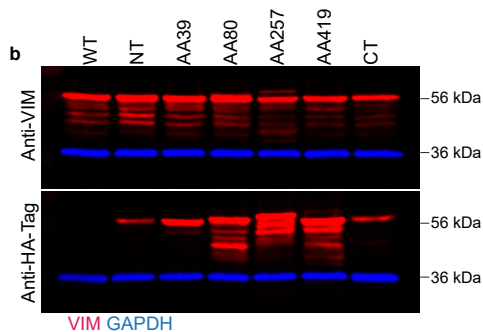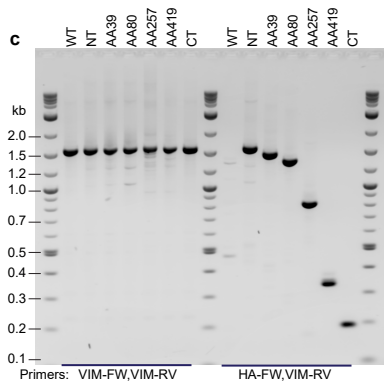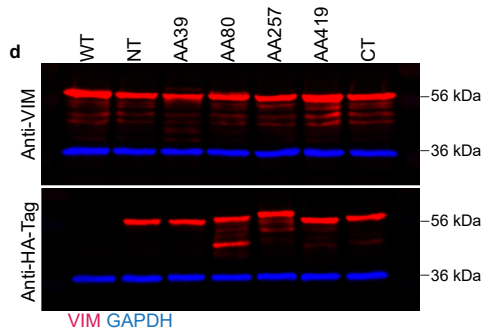

Supplement: Multimedia component 6 [file mmc6.pdf]

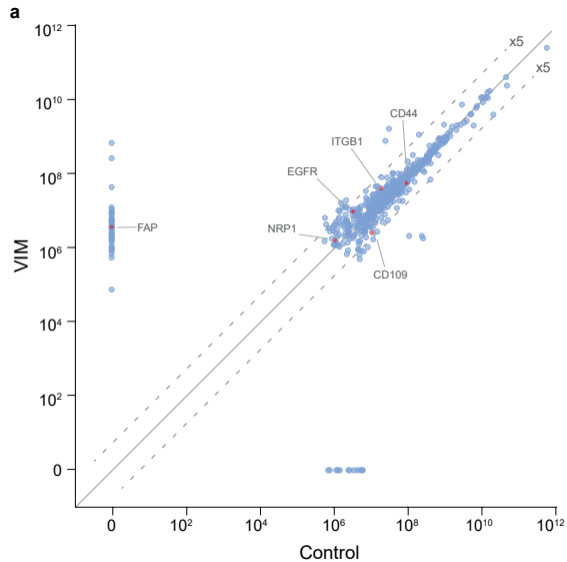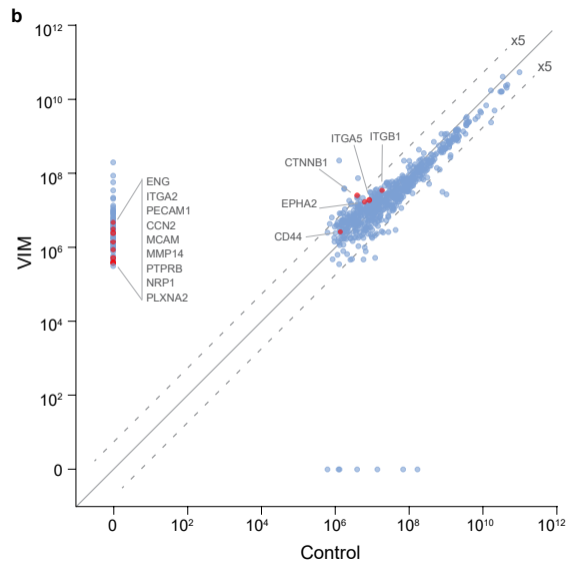

Supplement: Multimedia component 7 [file mmc7.pdf]

**a**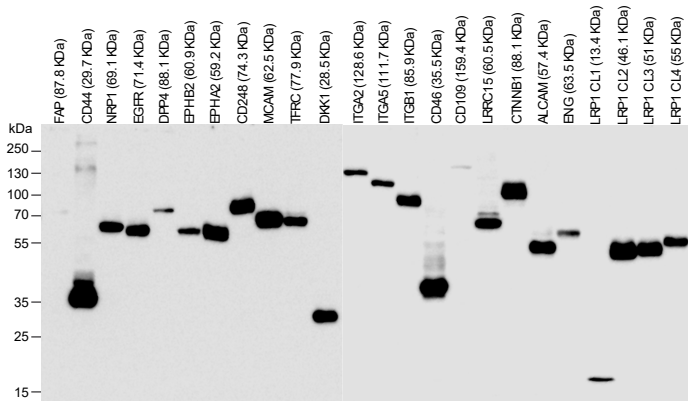**b**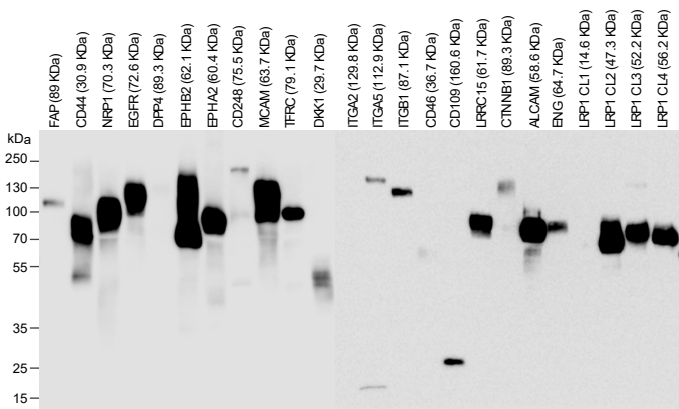**c**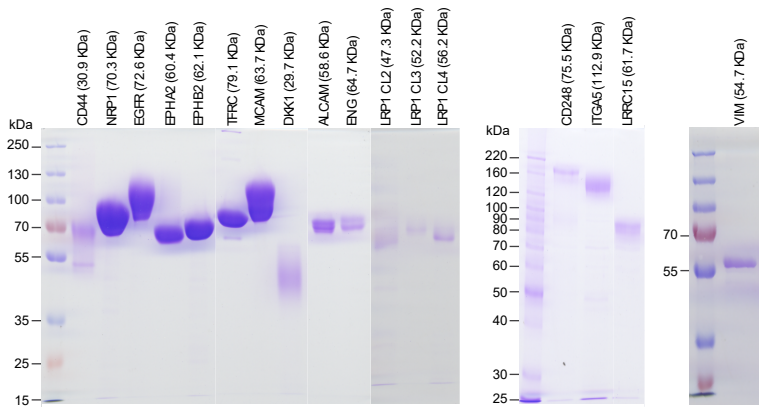

Supplement: Multimedia component 9 [file mmc9.pdf]
