# Supplementary material for: An adaptable genomic-proteomic approach to characterize cell-surface Vimentin's membrane topology and interactome
Source: Biochem Biophys Rep. 2025 Sep 6;44:102242. doi: 10.1016/j.bbrep.2025.102242 (PMC12447900; doi:10.1016/j.bbrep.2025.102242)
Supplement: Multimedia component 8 [file mmc8.pdf]

AP-MS Hit ID	RT-PCR	Purified	ELISA-VIM as Analyte	ELISA-VIM as Ligand
FAP	YES	NO		
CD44	YES	YES	–	–
NRP1	YES	YES	–	–
EGFR	YES	YES	–	–
DPP4	YES	NO		
EPHB2	YES	YES	Positive	Positive
EPHA2	YES	YES	Positive	Positive
CD248	YES	YES	–	–
MCAM	YES	YES	–	–
TFRC	YES	YES	Positive	Positive
DKK1	YES	YES	–	Positive
ITGA2	YES	NO		
ITGA5	YES	YES	Undecided	–
ITGB1	YES	NO		
CD46	YES	NO		
CD109	YES	NO		
LRRC15	YES	YES	Positive	–
CTNNB1	YES	NO		
ALCAM	YES	YES	–	–
ENG	YES	YES	–	–
LRP1 CL1	YES	NO		
LRP1 CL2	YES	YES	–	–
LRP1 CL3	YES	YES	–	–
LRP1 CL4	YES	YES	–	–
CSPG4	NO			
